# Unusual Sperm Morphology in Two Sedentary Songbird Species

**DOI:** 10.1002/ece3.71873

**Published:** 2025-07-30

**Authors:** Emily R. A. Cramer, Gaute Grønstøl, Phred M. Benham, Carla Cicero, Rauri C. K. Bowie, Daniel J. Tobiansky, Jan T. Lifjeld

**Affiliations:** ^1^ Natural History Museum University of Oslo Oslo Norway; ^2^ Marine Sciences, Tjärnö Marine Laboratory University of Gothenburg Strömstad Sweden; ^3^ Museum of Vertebrate Zoology University of California Berkeley Berkeley California USA; ^4^ Department of Integrative Biology University of California Berkeley Berkeley California USA; ^5^ Department of Biology St. Mary's College of Maryland St. Mary's City Maryland USA; ^6^ Program in Neuroscience St. Mary's College of Maryland St. Mary's City Maryland USA

**Keywords:** cryptic female choice, evolutionary jumps, female promiscuity, post‐copulatory sexual selection, sperm competition, weak selection

## Abstract

Sperm morphology can differ dramatically among closely related species. Within songbirds, the typical filiform sperm has a slender, corkscrew‐shaped head and an elongated midpiece coiled around the flagellum. However, three songbird species are known to have an unusual tadpole‐like sperm morphology with a round or ellipsoid head and short, uncoiled midpiece, which may arise developmentally via neoteny. Here, we describe tadpole‐like sperm phenotypes from two additional songbird species, the white‐breasted nuthatch ( *Sitta carolinensis* ) and the wrentit ( *Chamaea fasciata* ). These five species with tadpole‐like sperm share several ecological characteristics that can inform hypotheses for the evolution of this unusual phenotype: They are largely non‐migratory, form long‐lasting pair bonds, and have high paternal investment, small testes (no data for wrentit), and short and highly variable sperm lengths. These characteristics could indicate particular natural selection pressures driving physiological states such as low testicular testosterone levels. Additionally, though direct measures of female promiscuity are lacking in these species, these characteristics are consistent with weak post‐copulatory sexual selection. Further study of these similar yet independent evolutionary events, across all levels of analysis, would be valuable for understanding how such dramatic shifts in phenotype evolve.

## Graphical abstract

We describe two dramatic evolutionary shifts in sperm morphology in songbirds, which are independent of but similar to two previously known shifts. Species with this atypical, “tadpole‐like” sperm morphology appear to share sedentary life histories with long‐term pair bonds, high paternal investment, and likely low post‐copulatory sexual selection.

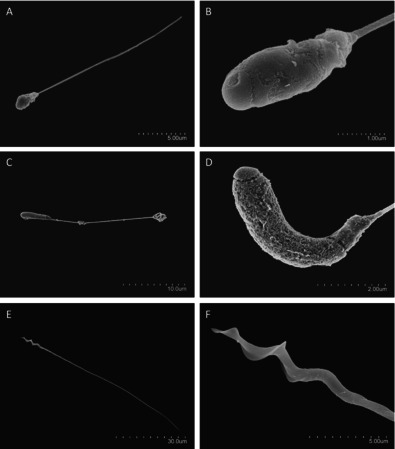

## 1 Introduction

Closely related organisms often exhibit similar phenotypes. Deviations from this typical pattern of phylogenetic conservatism, such as the dramatically different body plan of turtles compared to other amniotes, have long been a topic of interest and debate in evolutionary biology (Theißen 2006). Understanding how such large phenotypic shifts occur is crucial for gaining insights into the evolution of novelty. It is therefore essential to identify when and where in the phylogeny these significant and sudden phenotypic shifts have taken place. Independent yet similar phenotypic shifts may provide particularly powerful opportunities to understand evolutionary processes.

Dramatic shifts in the body plan of sperm cells are noted in several vertebrate lineages. For example, the sperm from mormyroid fish are unique among vertebrates in lacking flagella (Koenig and Gallant 2021). The loss of the flagellum likely renders Mormyrid sperm immotile, requiring behavioral compensation to ensure good egg‐sperm contact in these external fertilizers. It is hypothesized that the loss of the flagellum may reduce total metabolic costs and help offset the costs of electrogenesis in this group (Koenig and Gallant 2021). Additionally, muroid rodent sperm typically have a long apical hook, but in several instances, the hook has been lost within specific species or groups (Breed 2005). An in‐depth investigation of three muroid species, where the hook appears to have been lost recently and independently, reveals substantially elevated variation in head shape and in the length of the sperm tail (Breed et al. 2007). In both the fish and rodents, low post‐copulatory sexual selection is hypothesized as a key factor in the evolution of the novel sperm types (Breed 2005; Breed et al. 2007; van der Horst and Maree 2014; Koenig and Gallant 2021).

Dramatic shifts have also been observed in the songbird parvorder Passerida sensu Sibley and Ahlquist (1990). Spermatozoa of approximately 14% (580 of ~4000) of Passerida species have been examined and measured according to the SpermTree database (Fitzpatrick et al. 2022, and our own unpublished data from the avian sperm collection at the Natural History Museum, University of Oslo; Lifjeld 2019). Sperm cells in this parvorder are typically quite slender and helical, with acrosomes that are substantially longer than the nuclei. Generally, a helical membrane projects prominently from the acrosome, the nucleus is helical, and the midpiece consists of a long, fused mitochondria that coils around a large portion of the flagellum's length (Jamieson 2007). Helicity in the head and midpiece may help generate forward thrust in the unique form of motility observed in Passerida, whereby sperm swim by spinning around their longitudinal axis without appreciable bending in the flagellum (Vernon and Woolley 1999; Støstad et al. 2018). Other Passeriformes species show reduced helical membranes, with acrosomes generally shorter than the nucleus and midpieces often too short to observe helicity (Jamieson 2007). In other birds (except some Charadriiformes), acrosomes have no helical membrane, heads are only gently curved but not helical, and midpieces are generally short and fully encircle the flagellum (Retzius 1909; Jamieson 2007; Fitzpatrick et al. 2022). Most other bird groups are thus described as having “saurapsid” sperm forms. In stark contrast to other Passerida species, and indeed to birds in general, are the sister species Eurasian and Azores bullfinches ( *Pyrrhula pyrrhula* and *P. murina* , respectively) and the nominate subspecies of the Australian red‐browed finch ( *Neochmia temporalis temporalis* ; hereafter simply red‐browed finch). In these species, sperm cells have a “tadpole‐like” appearance without notable helical shaping and with heads proportionally much wider relative to the flagellum compared to other birds (Birkhead et al. 2006; Lifjeld et al. 2013; Rowe et al. 2025). These species' sperm are also short, with a short midpiece, and there is unusually high variation in sperm morphology within single ejaculates (Birkhead et al. 2006; Lifjeld et al. 2013; Rowe et al. 2025).

Studies that have identified shifts in sperm body plan have hypothesized that the simpler tadpole‐like phenotype occurs due to neoteny (at the proximate level of analysis) and because low post‐copulatory sexual selection reduces selective pressure on sperm quality in these taxa (at the ultimate level of analysis; Birkhead et al. 2006, 2007; Birkhead and Immler 2007; Van Der Horst et al. 2011; Lifjeld et al. 2013; Rowe et al. 2025). The spermatozoon of the Eurasian bullfinch has been described as appearing similar to a mid‐stage spermatid in overall structure (Aire 2014). Low post‐copulatory sexual selection is inferred from relatively small testes compared to body size and relatively high variation in sperm length measures among males in both avian and mammalian examples (Birkhead et al. 2006; Breed et al. 2007; Lifjeld et al. 2010, 2013; Rowe et al. 2025). An alternative proximate‐level hypothesis suggests that the tadpole‐like phenotype arose due to low genetic variation or inbreeding. However, this hypothesis has been rejected in the two bird species where it was tested (Durrant et al. 2010; Rowe et al. 2025).

The hypotheses that shifts in sperm phenotype have a neotenic origin and persist due to weak post‐copulatory sexual selection are intuitively appealing and align well with broader examples and theoretical frameworks regarding large phenotypic shifts (Bateman and DiMichele 2002). However, to rigorously evaluate this—or any—hypothesis, it is essential to generate and test alternative explanations (Platt 1964), ideally at multiple levels of analysis (Tinbergen 1963).

Sperm phenotypes are also shaped by natural selection, a factor that has been relatively underexplored in evolutionary studies (Reinhardt et al. 2015; Lüpold and Pitnick 2018). Natural selection may therefore provide additional hypotheses to explain shifts in sperm morphology. For example, the tadpole‐like sperm phenotype might evolve in response to low availability of antioxidants, which play a critical role in maintaining sperm membrane integrity and are present both in seminal fluid and in female sperm storage organs in birds (Bréque et al. 2003). Since the helical acrosomal membrane typical of passerine birds appears particularly vulnerable to oxidative damage (Støstad, Rowe, Johnsen, and Lifjeld 2019), species with limited antioxidant availability might experience selection against such a membrane. Antioxidant availability, in turn, may be influenced by diet and/or mitochondrial physiology. For example, long‐distance migration imposes substantial oxidative challenges for birds due to the high energy demand and the use of fat to meet those demands (McWilliams et al. 2021). Consequently, mitochondrial and antioxidant physiology may be under strong selection in migratory species (McWilliams et al. 2021).

In this paper, we describe two previously undocumented cases of tadpole‐like spermatozoa in songbirds. To identify potential hypotheses for the evolution of this phenotype, we compare ecological and life history traits among the songbird species that exhibit this sperm morphology. If factors such as diet, migratory behavior, or other ecological traits influence the evolution of the tadpole‐like sperm phenotype, we predict that species with tadpole‐like spermatozoa will share such traits.

## 2 Methods

### 2.1 Field Methods

We first observed white‐breasted nuthatch ( *Sitta carolinensis* ) sperm from one individual during a sampling trip targeting sperm of diverse species in May 2022. In 2023, we specifically targeted white‐breasted nuthatches while also sampling sperm of other species, including the wrentit ( *Chamaea fasciata* , *n* = 1). Fieldwork was conducted at three locations: Lassen National Forest, Lassen County, California (higher‐elevation woodlands in the Sierra Nevada Mountains, May 26–30, 2022, *n* = 1 white‐breasted nuthatch); Blue Oak Ranch Reserve (BORR), Santa Clara County, California (oak‐savannah habitat, March 31—April 1, 2023, *n* = 7 white‐breasted nuthatches and 1 wrentit); and at several locations within St. Mary's County, Maryland (deciduous forests, March 21–24, 2023, *n* = 4 white‐breasted nuthatches). Geographic coordinates for each individual are in the data repository (Cramer et al. 2025). Birds were collected by mistnetting (euthanasia by isoflurane overdose in California or general isoflurane anesthesia followed by rapid decapitation in Maryland) or shotgun under federal, state, and local permit authorizations. California birds were accessioned and cataloged in the Museum of Vertebrate Zoology, University of California, Berkeley (MVZ; catalog numbers in the data respository); Maryland samples also were used in DJT's ongoing neurobiology research at St. Mary's College of Maryland and Providence College. Sperm samples (California birds) or images (Maryland birds) were accessioned into the Natural History Museum of the University of Oslo (NHMO).

In 2023, we weighed testes of white‐breasted nuthatches with a digital jewelry scale precise to 1 mg (Smart weigh GEM20). Body mass was weighed with digital scales. Relative testes mass was calculated as the weight of both testes divided by body mass. Sperm samples were collected by cloacal massage or by dissecting from the seminal glomera and, for one white‐breasted nuthatch, the vas deferens (as the seminal glomera were difficult to find). The source of sperm for each sample is provided in the data repository (Cramer et al. 2025). We are confident that all sampled cells are morphologically mature because sperm are stored in the seminal glomera immediately prior to ejaculation, and we observed that sperm were swimming for many of the sampled individuals. Furthermore, studies of other bird species (red‐winged blackbirds, *Agelaius phoeniceus* ; Lüpold et al. 2011) have demonstrated that sperm collected from the seminal glomera do not show a different morphology from those collected by cloacal massage. Samples were diluted in phosphate‐buffered saline before being transferred to 5% formaldehyde.

Genomic evidence in white‐breasted nuthatches suggests that there are four highly differentiated populations that currently group into different subspecies, at least some of which may best be considered species (Askelson et al. 2023). We sampled two of these subspecies, *S. c. carolinensis* from Maryland (Eastern clade) and *S. c. aculeata* from BORR (Pacific clade). Our sample from Lassen County falls within a region of contact between *S. c. aculeata* and *S. c. tenuissima* of the Rocky Mountain North clade, although unpublished whole genome data (Arulanantham et al.) show this individual clustering with other samples of *S. c. tenuissima*. These two subspecies are vocally distinct (Pandolfino et al. 2017), but we did not hear the vocalization of our sampled individual. In light of these geographic differences, we show results separately by sampling locality.

### 2.2 Sample Analyses

We pipetted approximately 15 μL of fixed sperm cells onto glass slides for light microscopy. After the slides dried overnight, we rinsed them with distilled water and imaged cells at 320× magnification following Grønstøl et al. (2023). We further examined slides using phase‐contrast settings, which did not substantially differ in quality (e.g., ability to distinguish morphological detail) compared to images from brightfield microscopy settings. From brightfield microscopy images, we measured the length of the head (acrosome and nucleus), midpiece, and exposed flagellum using Leica Application Suite X v. 3.7.2 (Leica Microsystems, Switzerland). Flagellum length was calculated as the sum of the midpiece and exposed flagellum, and total sperm length was the sum of the head and flagellum. We chose only complete, normal cells to measure (where normal is defined as having a smooth head shape rather than a sharp edge that could reflect acrosome reaction, for example, Støstad, Rowe, Johnsen, Tomášek, et al. 2019, and with the flagellum tapering toward the distal end). Following Laskemoen et al. (2007), we aimed to measure 10 cells per individual. Due to logistical constraints with human resources and sample export for Maryland samples, we could not measure 10 cells for all these individuals (5–10 cells per male measured, mean of 7.5 ± 2.4 SD; see data repository). We calculated the within‐male coefficient of variation in total sperm length (CV_wm_) using all measured cells, as well as among‐male coefficient of variation in total sperm length (CV_am_) using the mean total sperm length from each male. CV was calculated as 100*SD/mean and corrected for low sample size by multiplying by a factor of (1 + 1/(4*n*)), where *n* is the number of cells or males, respectively (Sokal and Rohlf 1995).

Samples from three individuals (one white‐breasted nuthatch from Lassen County, one white‐breasted nuthatch from BORR, and one wrentit from BORR) were prepared for scanning electron microscopy following Cramer et al. (2022). Briefly, samples were dehydrated in ethanol, subjected to critical point drying (i.e., replacing the ethanol with liquid carbon dioxide and letting the carbon dioxide evaporate), and sputter‐coated with platinum. For comparison, we obtained scanning electron micrographs of two Eurasian and one Azores bullfinch samples from the NHMO avian sperm collection (Lifjeld 2019). One Eurasian bullfinch and the Azores bullfinch were described in Lifjeld et al. (2013) and images of the other Eurasian bullfinch were provided by Martin Oeggerli (pers. comm.). From these images, we measured the dimensions of the nucleus and the acrosome following Støstad et al. (2018) as closely as possible, given the dramatic difference in shapes between these species and “typical” passerine sperm. We calculated acrosome volume as a cone (Støstad et al. 2018). We further measured the diameter of the flagellum immediately after the end of the midpiece following Cramer et al. (2022) in ImageJ (Schneider et al. 2012). Acrosome volumes were measured by GG and flagellum diameters by ERAC, without removing information on sample or species identity. We aimed to measure acrosome and nucleus volume for five cells per male. This target was chosen for a larger macroevolutionary analysis where we needed to balance within‐species precision against measuring many species. Due to low sperm count on the SEM stubs (due to cell loss during preparation steps), the mean ± SD number measured was 3.8 ± 1.6 (range 1–5; see data repository). Flagellum diameters were measured opportunistically from SEM images taken for head and whole‐cell image purposes (4.8 ± 1.7, 2–7 cells; see data repository).

### 2.3 Additional Data and Statistical Tools

For comparison, we obtained data on the length of the sperm head, midpiece, and flagellum from other bird species. For Passeriformes, we used the average of all measurements from the NHMO collection (*n* = 7061 accessions and 649 species measured as of November 13, 2024). For non‐Passeriformes, we used data from the NHMO and from SpermTree (Fitzpatrick et al. 2022), averaging values when a species was represented in both datasets (*n* = 89 species). Data on the red‐browed finch are from Rowe et al. (2025). We used only the NHMO data for Passeriformes because individual‐level data were available for calculating CV_wm_, sample sizes were readily available for CV_am_, and we could ensure consistent measurement of midpiece (as other research groups sometimes use a different measurement of the Passeriformes' mitochondrial helix, e.g., Durrant et al. 2020). For species with multiple populations sampled, CV_am_ was calculated separately by populations to avoid inflating among‐male variation with between‐population mean sperm length differences. Only populations with at least eight males were included for CV_am_.

To contextualize relative testes size, we used data from the Passerides clade of Passeriformes sourced from Calhim and Birkhead (2006), Calhim and Montgomerie (2015), and Rowe et al. (2015). When species appeared in multiple datasets, we averaged the values. In summarizing relative testes size across species with typical sperm, we removed the Eurasian bullfinch and white‐breasted nuthatch (since they have tadpole‐like sperm) and the white‐throated dipper ( *Cinclus cinclus* , which was an order of magnitude smaller than the next‐lowest value and was measured from the pre‐breeding months). Because bird testes are much reduced in size and do not produce sperm outside the breeding season, it is necessary to compare them during the breeding season when they are actively producing sperm (Calhim and Birkhead 2006). Although we are unsure about the exact breeding stages of the individuals we sampled because we did not locate nests, the testes were mature by definition because sperm were being released.

In addition, we searched for data on extra‐pair paternity rates for the white‐breasted nuthatch and wrentit (Brouwer and Griffith 2019), as extra‐pair paternity reflects female promiscuity and the opportunity for post‐copulatory sexual selection (Cramer et al. 2020). To compare natural history characteristics across species, we drew primarily on the species' accounts in Handbook of Birds of the World (Clement and Christie 2020; Clement et al. 2020; Grubb Jr and Pravosudov 2020; Payne 2020).

After identifying possible commonalities among species with tadpole‐like sperm, we statistically tested for associations between these traits and sperm form. We performed these tests at the family level for three reasons, scoring each family as having (or not having) at least one species with tadpole‐shaped sperm and using a composite value for the ecological/life‐history trait. First, extra‐pair paternity data are only available at the family level, so performing all tests at this level facilitates comparing test results across hypotheses. Second, the imbalance in sample size between typical and tadpole‐like sperm groups is ameliorated. Third, as we describe in the discussion, we reason that mutations that generate the phenotype are likely rare and arise randomly across the phylogeny; ecological and life‐history traits may impact whether the tadpole‐like phenotype can persist but not where they arise. Thus, many species are expected to have traits that would allow tadpole‐like sperm to persist, without having tadpole‐like sperm, simply because such a mutation has not occurred in their lineage. Including such lineages reduces statistical power, especially in a species‐level analysis where they will be numerically much more dominant.

We obtained data from already‐compiled sources and only filled in missing data for species with tadpole‐like sperm. For relative testes size, migration distance, and extra‐pair paternity rate, we used the average values across the taxonomic family from the following sources. Relative testes size and extra‐pair paternity were as described above (Calhim and Birkhead 2006; Calhim and Montgomerie 2015; Rowe et al. 2015; Brouwer and Griffith 2019). Migration distance (in 1000 km) was obtained from Lifjeld et al. (2019) and reflects the straight‐line distance from their study population to the center of the winter distribution, with partial migrants and Afrotropical species assigned a 0 value under the assumption that migration distance averages less than 500 km. Early‐stage male parental investment was from Gonzalez‐Voyer et al. (2022) and pair bond duration was from Tobias et al. (2016). We summarized the data by calculating the proportion of species with male investment greater than 0 in either the nest building or incubation categories, or with a long‐term pair bond (category 3 in Tobias et al. 2016). We reconciled species names to the IOC 11.2 taxonomy (Gill et al. 2024) using Avonet (Tobias et al. 2022) and Avibase (Lepage et al. 2014). To account for phylogenetic relatedness, we trimmed the phylogeny produced in Stiller et al. (2024) to contain one representative of each relevant family using the keep.tip function (package ape, Paradis et al. 2004).

Finally, we created a separate model for each hypothesis, with sperm form as the response variable and the family‐level composite ecological/life history measure as the predictor. We then used the threshBayes function in phytools (Revell 2012, 2016) to evaluate whether the variables were related. Using the logic of Felsenstein et al. (2012), this function assumes that binary variables change state depending on whether an unobserved, continuous “liability” variable is above or below a threshold. The correlation between the liability variables of two binary variables or between one binary and one continuous variable is then estimated. We ran 1,000,000 MCMC generations and estimated the effective sample sizes using the effectiveSize function in the package coda (Plummer et al. 2006).

To investigate phylogenetic history at the species level, we created a consensus tree from 1000 stage 2 trees downloaded from Birdtree.org (Jetz et al. 2012; Ericson backbone, trimmed to the species of interest using the keep.tips tool). We found the mean branch length using TreeAnnotator (Drummond and Rambaut 2007; Bouckaert et al. 2019) and removed species where negative branch lengths resulted (all distant from species with tadpole‐shaped sperm). Using the function ancThresh (package phytools, Revell 2012, 2016), which uses the same logic and liability variable approach as the threshBayes function, we reconstructed the evolution of tadpole‐shaped sperm across all Passerida species with known sperm form.

Data were summarized and analyses were conducted in R v 4.2.3 (R Development Core Team 2012) using the following packages: tidyverse (Wickham et al. 2019), ggplot2 (Wickham 2016), ape (Paradis et al. 2004), cowplot (Wilke 2020), ggtree (Yu 2020), and tidytree (Yu 2022). Raw data on the white‐breasted nuthatch and wrentit are provided in Cramer et al. (2025).

### 2.4 Notes on Sample Size, Breeding Condition, and Typical Ejaculates

While sampling sperm from only one male is not ideal and does not allow for statistics, we find it unlikely that the wrentit sperm we describe here is unusual for the population. Our group has examined sperm from 6898 males representing 431 species for which the NHMO has samples from two or more males, and in no case do we find intra‐population variation in sperm body plan; the red‐browed finch is the only known case of intra‐specific variation (between subspecies). Therefore, it is more likely that we sampled a typical wrentit sperm rather than sampling a species‐atypical phenotype for the single wrentit male that we captured. Furthermore, it is also not likely that immature sperm are released from the testes before the sperm‐producing machinery is fully developed. We have taken cloacal massage samples from several passerines before the start of the breeding season over > 20 years of fieldwork, and these samples either contain typical sperm or no sperm but not immature cells.

### 2.5 Ethical Approvals

Ethical and legal permits were as follows: Maryland Department of Natural Resources Scientific Collecting permit 58161 to DJT; US Fish and Wildlife Service Scientific Collecting permit MBPER0039765 to DJT; US Fish and Wildlife Service Scientific Collecting permits MB153526‐0 (with extension) and MBPER0040862 to RCKB and CC; California Department of Fish and Wildlife scientific collecting permit SCP‐458 (with extension) to RCKB and CC; Institutional Animal Care and Use Committee approvals R112101 to DJT (St. Mary's College of Maryland), AUP‐2015‐10‐8045‐3 to CC (University of California, Berkeley), and AUP‐2016‐04‐8665‐2 to RCKB (University of California, Berkeley).

## 3 Results

**FIGURE 1:**
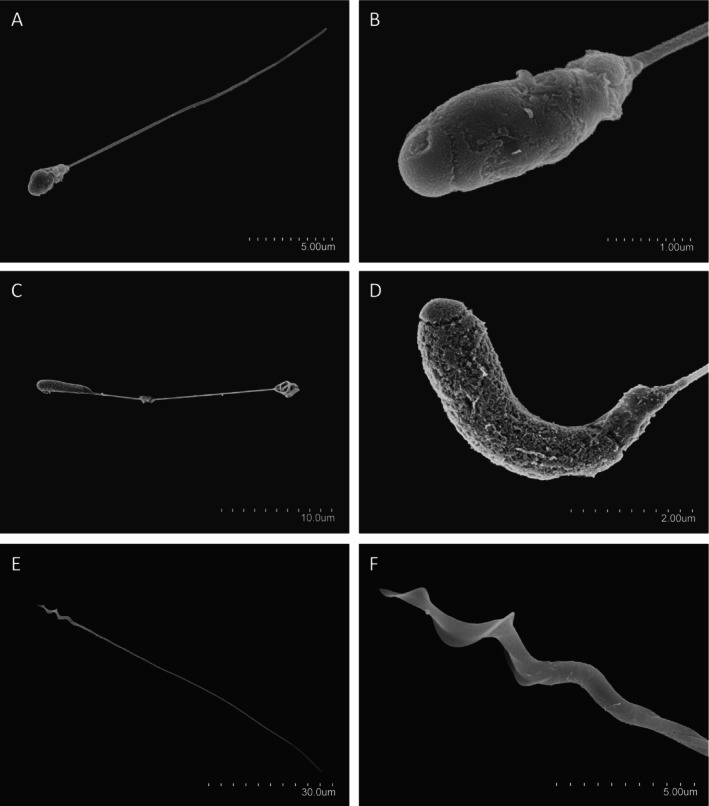
Sperm cells (A, C, E); the head and midpiece of sperm cells (B, D) or head only (F) of two species with tadpole‐like sperm (A–D) and of a typical Passerida sperm (E, F), for comparison. Panels (A) and (B) are white‐breasted nuthatch; (C) and (D) wrentit; and (E) and (F) dunnock, *Prunella modularis* . Images were taken with scanning electron microscopy, and scale is indicated separately by image panel. Note that the wrentit flagellum (panel C, right side of cell) appears to be abnormally coiled. Image backgrounds were modified for consistent contrast and to remove debris.

Sperm from all white‐breasted nuthatches (*n* = 11) and the wrentit (*n* = 1) had a tadpole‐like phenotype (Figure 1). We observed sperm swimming from cloacal massage and seminal glomera samples from the Lassen white‐breasted nuthatch, from all four Maryland males, and from one of the three BORR males. Swimming of sperm was also confirmed from the vas deferens sample of the wrentit (morphology similar to sperm from the the seminal glomera; Cramer et al. 2025).

**FIGURE 2:**
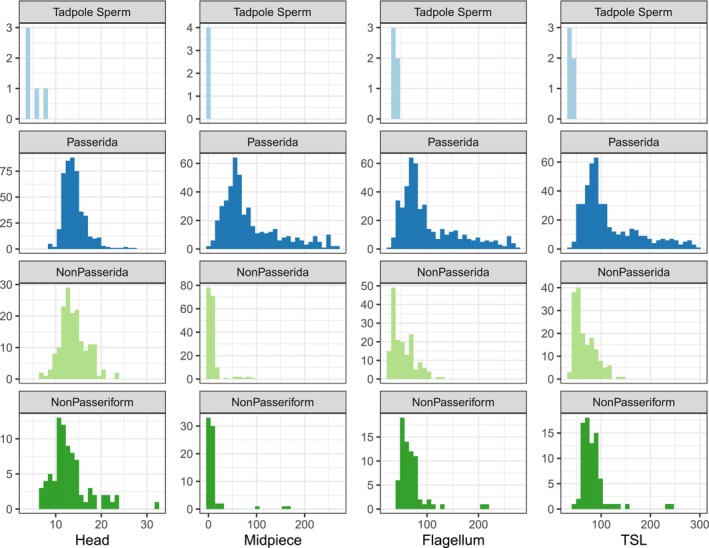
Histograms comparing the length of sperm components (from left to right: Head, midpiece, flagellum, and total sperm length [TSL], in μm) from species with tadpole‐like sperm (top), their closest relatives (the parvorder Passerida), other Passeriformes outside parvorder Passerida (NonPasserida), and birds outside Passeriformes (NonPasseriform, bottom). Data from Passeriformes comes from the NHMO only, and data on non‐Passeriform species is from the NHMO and SpermTree (Fitzpatrick et al. 2022).

**FIGURE 3:**
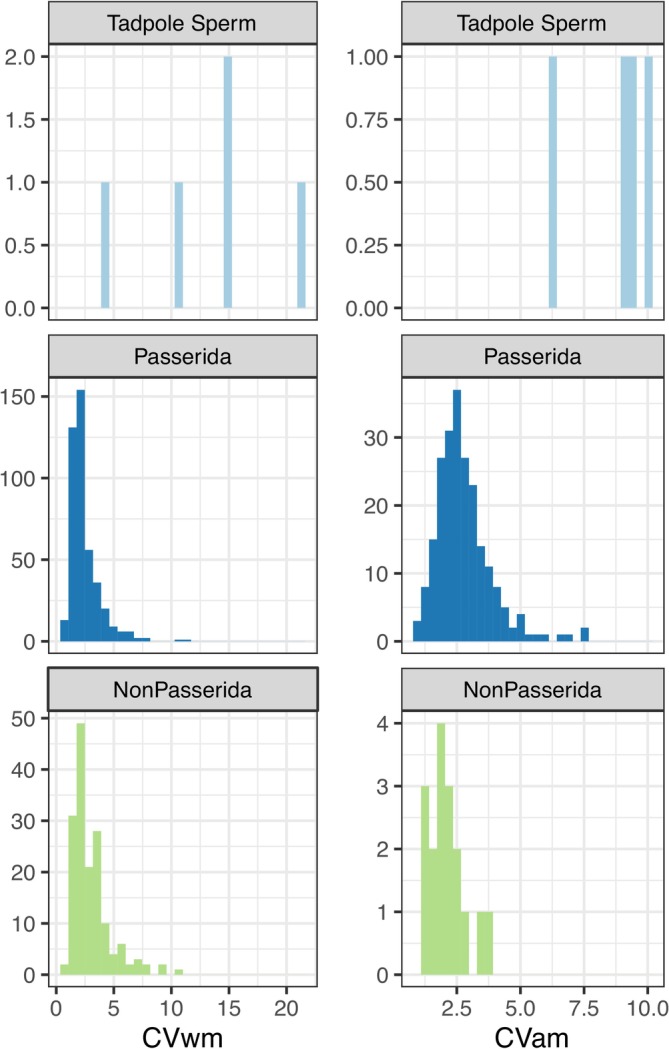
Histograms comparing within‐male (left, CVwm) or among‐male (CVam) coefficient of variation in sperm total length (units: %). Data from species with tadpole‐like sperm (top), their closest relatives (the parvorder Passerida), and other Passeriformes outside parvorder Passerida (NonPasserida) are included. For CVam, we calculated the coefficient of variation separately for each island, country, or named subspecies to avoid conflating between‐population divergence with within‐population variation. Only populations with at least eight males were included for CVam. Data are from the NHMO.

Sperm from the white‐breasted nuthatch and wrentit were short and highly variable in length, like other bird species with tadpole‐like sperm (Eurasian bullfinch, Azores bullfinch, red‐browed finch), but are markedly different from members of the parvorder Passerida (Figures 2 and 3, Table 1). The sperm head length is dramatically shorter than in Passerida and other birds more broadly (Figure 2). The bullfinches, white‐breasted nuthatches, and wrentit acrosomes were small (Table 2) but not extreme compared to species measured by Støstad et al. (2018). Nucleus volumes were above average, with the wrentit exceeding the largest nucleus volume of all species included in the Støstad et al. (2018) dataset (Table 2). The white‐breasted nuthatch sperm head appeared roughly spherical (similar to the Eurasian and Azores bullfinches), whereas the wrentit sperm head was more of an elongated ellipsoid (similar to the red‐browed finch; Figure 1). The flagellum of the white‐breasted nuthatch and wrentit sperm is slender and comparable to that of the bullfinches (Table 2). Total sperm length for both species was substantially shorter than the closest relatives with available data (white‐breasted nuthatch, 36.8 ± 4.21 across all 11 males, compared to 77.95 ± 2.94 [European nuthatch *Sitta europaea* , *n* = 11] and 75.85 ± 5.35 [red‐breasted nuthatch, *S. canadensis* , *n* = 3]; wrentit 37.94, compared to 82.75 ± 6.84 across 306 males of 13 species in family Sylviidae [genera *Sylvia* and *Curruca* only]).

**TABLE 1:** Mean length of sperm components across males and coefficients of variation within males (CV_wm_) or among males (CV_am_) from white‐breasted nuthatch (WBNU) and wrentit, with other species for comparison.

Species and locality	Head (μm)	Midpiece (μm)	Flagellum (μm)	Total length (μm)	CV_wm_ (%)	CV_am_ (%)
WBNU eastern clade (Maryland; 5–10 cells per male, 4 males)	4.26 ± 0.69	1.15 ± 0.09	34.83 ± 5.74	39.10 ± 5.98	19.14 ± 6.25	16.4
WBNU Pacific clade (BORR; 10 cells per male, 7 males)	3.20 ± 0.34	1.44 ± 0.16	32.29 ± 2.44	35.49 ± 2.47	13.70 ± 6.26	7.2
WBNU contact zone (Lassen, 10 cells, 1 male)	3.05	1.27	34.20	39.01	5.73	N/A
Wrentit (10 cells, 1 male)	6.15	1.73	32.63	38.78	20.98	N/A
Other tadpole‐like Passerida (means: *n* = 3 species; CV_wm_ *n* = 3, CV_am_ *n* = 3)	5.03 ± 2.05 (3.68–7.39)	1.73 ± 0.16 (1.61–1.84)	40.24 ± 7.36 (31.75–44.76)	45.28 ± 5.33 (39.14–48.79)	9.87 ± 5.37 (4.13–14.77)	8.42 ± 1.91 (6.29–9.96)
Typical passerida (means: *n* = 460 species, CV_wm_ *n* = 437, CV_am_ *n* = 222)	14.09 ± 2.57 (8.54–27.23)	87.20 ± 60.39 (1.90–269.16)	103.62 ± 57.73 (28.95–275.75)	117.71 ± 59.36 (39.08–293.10)	2.44 ± 1.32 (0.70–11.11)	2.78 ± 1.08 (0.86–7.64)
Non Passerida (means: *n* = 257 species, CV_wm_ *n* = 161, CV_am_ *n* = 17)	13.70 ± 3.48 (6.65–31.88)	10.46 ± 20.27 (1.24–165.00)	57.77 ± 25.13 (24.99–215.00)	72.18 ± 26.01 (31.88–240.00)	3.02 ± 1.70 (0.80–10.96)	2.10 ± 0.72 (1.30–3.87)

*Note:* These data are from light microscopy. A mean across cells within one male was calculated first before calculating the mean and SD among males shown here. For comparison, we provide mean ± SD (range) for other Passerida species with tadpole‐like sperm, from other Passerida species, and from other birds outside Passerida.

**TABLE 2:** Volume of acrosome and nucleus, and flagellum diameter at the end of the midpiece, for tadpole‐like sperm, measured from scanning electron microscope images for individual males (NHMO accession numbers provided).

Species (accession #)	Acrosome volume (μm^3^; *n* cells)	Nucleus volume (μm^3^; *n* cells)	Flagellum diameter (μm; *n* cells)
Azores bullfinch^a^	0.43 ± 0.12 (*n* = 5)	2.46 ± 0.78 (*n* = 5)	0.17 ± 0.02 (*n* = 5)
Eurasian bullfinch^a^	0.68 ± 0.14 (*n* = 5)	2.31 ± 0.66 (*n* = 5)	0.17 ± 0.01 (*n* = 5)
Eurasian bullfinch^b^ (106880)	0.40 ± 0.15 (*n* = 5)	2.48 ± 0.85 (*n* = 5)	0.17 ± 0.03 (*n* = 4)
White‐breasted nuthatch (contact zone, 108816)	0.33 (*n* = 1)	2.08 (*n* = 1)	0.14 ± 0.01 (*n* = 2)
White‐breasted nuthatch (Pacific clade, 110083)	0.32 ± 0.06 (*n* = 4)	2.73 ± 0.49 (*n* = 4)	0.13 ± 0.01 (*n* = 7)
Wrentit (110100)	0.58 ± 0.60 (*n* = 3)	4.34 ± 1.88 (*n* = 3)	0.15 ± 0.03 (*n* = 6)
Typical passerida (*n* = 36 species)^c^	1.87 ± 1.48 (range: 0.48–7.18, 36 species)	1.86 ± 0.35 (range: 1.44–3.23, 36 species)	

*Note:* Sample sizes differ for head and flagellum measurements due to image suitability for each cell component.

^a^ Images prepared for Lifjeld et al. (2013).

^b^ Images of an NHMO object provided by Martin Oeggerli.

^c^ Data from Støstad et al. (2018).

Relative testes mass (i.e., combined testes mass/body mass, expressed as a percentage) was 0.46% ± 0.13% for Eastern white‐breasted nuthatches (Maryland, *n* = 4) and 0.22% ± 0.04% for Pacific white‐breasted nuthatches (BORR, *n* = 6); we did not measure this for the wrentit. Previous work estimated relative testes mass for the white‐breasted nuthatch as 0.4% (Calhim and Montgomerie 2015). For comparison, relative testes mass for other species with tadpole‐like sperm is 0.22%–0.29% for Eurasian bullfinch (Lifjeld et al. 2013; Birkhead et al. 2006), and 0.21% for red‐browed finch (Rowe et al. 2025). For Passerida species with typical sperm, relative testes mass is 1.52% ± 1.09% (mean ± SD, range: 0.10%–6.45%; Calhim and Birkhead 2006; Calhim and Montgomerie 2015; Rowe et al. 2015).

**FIGURE 4:**
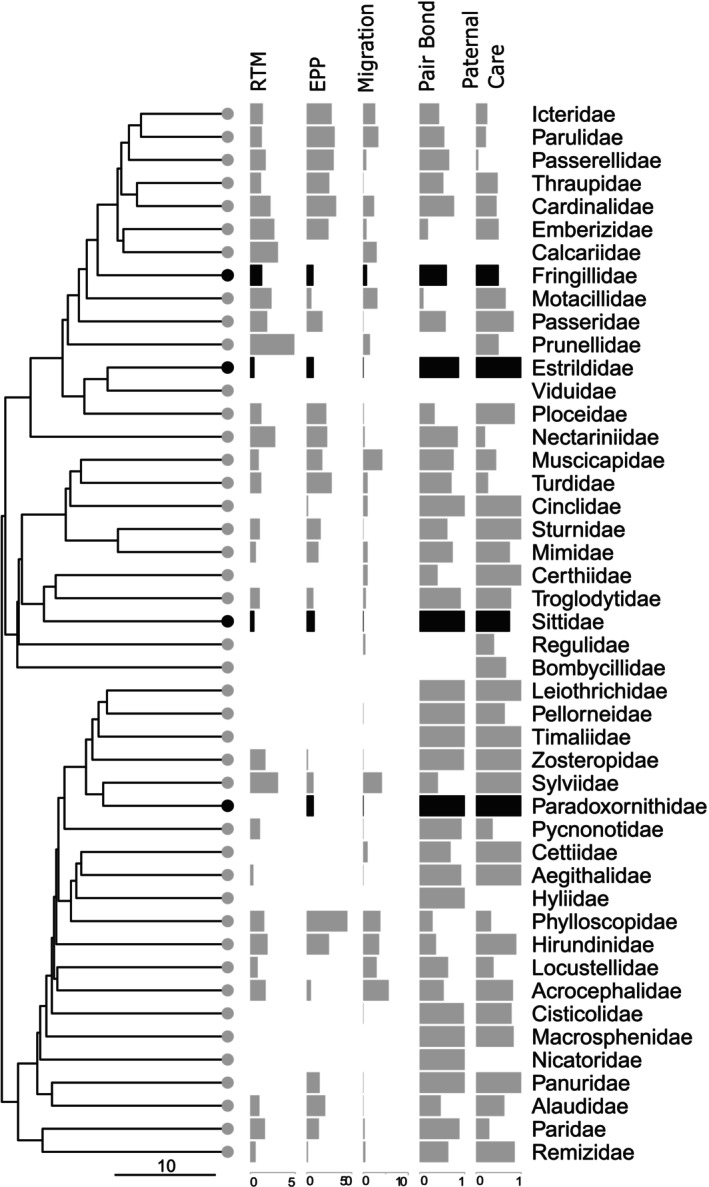
Family‐level Passerida phylogeny showing taxonomic families for which researchers at the UiO NHM Avian Sperm Collection have examined sperm from at least one individual. Families with a black dot at the tip and in bar graphs have at least one species with a tadpole‐shaped sperm, whereas families with gray dots have only slender, “typical” sperm body plans for all examined species. Summarized ecological/life‐history traits are relative testes mass (RTM, %); extra‐pair paternity rate (EPP, % extra‐pair offspring), migration distance (1000's of km), proportion of species with long‐term pair bonds, and proportion of species where males contribute to care during nest‐building and/or incubation states. Scale bar under the phylogeny shows 10 Myr. Only socially monogamous or polygynous species were included in the extra‐pair paternity dataset; many families have no data. An arbitrarily small value was added to families with very low values for EPP and migration distance to make it possible to visually distinguish missing data from low values.

We did not find studies of extra‐pair paternity in white‐breasted nuthatches nor wrentits. However, substantial variation in extra‐pair paternity occurs between taxonomic families, so we searched for socially monogamous species in Sittidae (relevant for white‐breasted nuthatches) and Paradoxornithidae (relevant for wrentits). Species in both families have relatively low extra‐pair paternity (Figure 4; Eurasian nuthatch 9.6% of offspring, Segelbacher et al. 2005; chestnut‐vented nuthatch *Sitta nagaensis* , 7.9%, Yuan et al. 2024; vinous‐throated parrotbill *Sinosuthora webbiana* , 7.7%, Lee 2012; mean ± SD rate for socially monogamous or polygynous species in parvorder Passerida 18.92% ± 13.64%, Brouwer and Griffith 2019).

### 3.1 Natural History

All five species with tadpole‐like sperm are largely non‐migratory, although the Eurasian bullfinch performs moderate‐distance seasonal migration in part of its range. Pair‐bonds last longer than one season in the three best‐studied species (Eurasian bullfinch, white‐breasted nuthatch, and wrentit; data are lacking for the Azores bullfinch and red‐browed finch; Hogstad 2016; Clement and Christie 2020; Clement et al. 2020; Grubb Jr and Pravosudov 2020; Payne 2020; Geupel and Ballard 2023). Paternal investment is also elevated for these three species, with males feeding females as the females incubate (Eurasian bullfinch and white‐breasted nuthatch; Clement and Christie 2020; Grubb Jr and Pravosudov 2020) or with males also incubating and brooding young (wrentit; Geupel and Ballard 2023).

In other respects, these five species appear somewhat dissimilar. Adult bullfinches and red‐browed finches rely primarily on plant‐based food, while the nuthatch and wrentit rely more heavily on insects, at least during the summer. The species have a range of nest shapes, including open‐cup, retort, and secondary cavities, and they exploit differing habitat types (although most require some trees or shrubs). Clutch sizes, which may impact sperm storage duration (Kleven et al. 2009), differ somewhat (2–3 for Azores bullfinch, Clement et al. 2020; 3–4 for wrentit, Geupel and Ballard 2023; mean of 6.4 for white‐breasted nuthatch, Grubb Jr and Pravosudov 2020; and 4–6 for Eurasian bullfinch and red‐browed finch, Clement and Christie 2020; Payne 2020). These values appear in line with expectations according to phylogeny and breeding range (Jetz et al. 2008). Body size, which may impact sperm size due to selective pressures associated with sperm dilution (Immler et al. 2011), ranges from about 11 to 30 g across the five species, and was similar to the body size of congeners (Tobias et al. 2022). Plumage coloration is similar between the sexes except for the Eurasian bullfinch, where males are brightly colored and females are drab (Hogstad 2016; Clement and Christie 2020; Clement et al. 2020; Grubb Jr and Pravosudov 2020; Payne 2020).

### 3.2 Family‐Level Analyses and Phylogenetic History

Relative testes mass, extra‐pair paternity rate, and migration distance were negatively, but nonsignificantly, associated with the occurrence of tadpole‐shaped sperm in family‐level analyses (highest posterior density [HPD] intervals barely including 0; Table 3, Figure 4). For long‐term pair bonds and male investment in early parental care, estimated correlations were more centered around zero, indicating less evidence for a relationship (Table 3).

**TABLE 3:** Estimated correlations between ecological/life‐history traits and the occurrence of at least one species with tadpole‐shaped sperm at the family level.

Traits	ESS	Correlation coefficient	
		Mean	HDP 95% interval
Relative testes mass	240.8	−0.50	−0.92, 0.02
Extra‐pair paternity	375.0	−0.44	−0.87, 0.03
Migration distance	124.3	−0.45	−0.91, 0.04
Proportion long‐term bonds	288.6	0.33	−0.12, 0.81
Proportion early male paternal investment	418.3	0.26	−0.20, 0.72

*Note:* The effective sample size (ESS) from the MCMC chain as well as mean and 95% highest posterior density (HPD) are given.

Tadpole‐shaped sperm evolved independently in each of the four taxonomic families where it appears, as the 95% HPD for the liability function did not include the threshold value (0) for common ancestors of the families. While parsimony strongly suggests that the tadpole‐like shape evolved before the split of the Eurasian and Azores bullfinches, the 95% HPD included 0, indicating that it is plausible that the immediate ancestor had typical‐shaped sperm (mean −1.35, range −4.56 to 2.57).

## 4 Discussion

Sperm morphology for two subspecies of white‐breasted nuthatch and for the wrentit appear very similar to the morphology of the Eurasian and Azores bullfinches and the red‐browed finch, all of which contrast strikingly with other species in the parvorder Passerida. Comparing these independent evolutionary events may therefore represent a powerful approach for understanding how the tadpole‐like sperm phenotype forms and persists. All five of the species are not migratory and have traits consistent with low female promiscuity, though promiscuity levels require direct testing. Further, where data are available, they have long‐term pair bonds and high paternal investment in their offspring. This suite of traits suggests the possibility of low post‐copulatory sexual selection and some similar natural selection pressures, which together may explain how these striking evolutionary shifts in sperm morphology can persist (though the onset of the phenotype may occur by rare, random mutations). We structure the discussion of hypotheses roughly around Tinbergen's non‐mutually exclusive levels of analysis (Tinbergen 1963).

### 4.1 Similarity to Other Tadpole‐Like Sperm Phenotype

As with the bullfinches and red‐browed finch, the white‐breasted nuthatch and wrentit have sperm with short, rounded, or ellipsoid heads that consist of a relatively small acrosome and a large nucleus. Large nucleus volume may be due to incomplete chromatin compaction (Lifjeld et al. 2013; Rowe et al. 2025). Flagella are slender in all species; in the bullfinches and red‐browed finches, transmission electron microscopy confirms that this slenderness is explained by the absence or substantial reduction in the diameter of the outer dense fibers of the axoneme (Birkhead et al. 2007; Lifjeld et al. 2013; Rowe et al. 2025). We hypothesize that these fibers are also reduced in the white‐breasted nuthatch and wrentit. Since chromatin compaction, acrosome elongation and elaboration, and outer dense fiber development occur relatively late in spermatogenesis (Aire 2014), all of these phenotypes are consistent with this phenotype being neotenous.

Outer dense fibers are thought to function partially in providing structural support to the cells (Lindemann and Lesich 2016), and we propose that the absence of the supportive components constrains flagellum length to be relatively short. Corroborating this hypothesis, an analogous supportive structure in mammals (the fibrous sheath) has been lost in two mammals with putative neotenous sperm and short flagella (the naked mole rat *Heterocephalus glaber* and greater bandicoot rat *Bandicota indica* ; Breed et al. 2007; Van Der Horst et al. 2011; Dorman et al. 2014). The fibrous sheath is, however, retained in a third species with putatively neotenous sperm (the spinifex hopping mouse *Notomys alexis* ), where the flagellum is not short but rather like close relatives (about 100 μm; Bauer and Breed 2006; Breed et al. 2007).

Small acrosome size may also have functional consequences, as low volume may reduce proteolytic enzymes that the acrosome can deliver as the sperm interacts with the egg's cell membrane (Dorman et al. 2014). Intriguingly, male Eurasian bullfinches appear unable to fertilize the eggs of canaries ( *Serinus canaria* ) or other fringillid finch species in captive crosses, although female bullfinches can be fertilized interspecifically (Birkhead and van Balen 2007).

### 4.2 Phylogenetic History

The tadpole‐like sperm phenotype evolved at least four times in Passeriformes (Figure 4). Parsimony suggests that the tadpole‐like phenotype arose prior to the diversification of the Azores and Eurasian bullfinches about 1 Myr ago (Töpfer et al. 2011) and prior to the diversification of the eastern and western clades of white‐breasted nuthatch about 1.6 Myr ago (Askelson et al. 2023), although formal analysis was inconclusive in the former case and not feasible in the latter (as we used a species‐level phylogeny). Thus, the phenotype can persist for hundreds of thousands of years.

The tadpole‐like phenotype in red‐browed finches (nominate subspecies) appears to have evolved within the last 1.2 Myr (the estimated age of the split from the other subspecies with typical sperm; Olsson and Alström 2020; Rowe et al. 2025). To evaluate the minimum plausible age of the wrentit phenotype, it would be interesting to sample wrentits from different genetic clades across the geographic range (Burns and Barhoum 2006). Given the putative species‐level divergence between eastern and western populations of white‐breasted nuthatches (Askelson et al. 2023), a comparison of sperm morphology between those genetic lineages would be interesting in the context of potential reproductive isolation; preliminary assessment suggests differences in sperm length, but further study based on additional samples is necessary. Sperm have neither been examined for the closest relative of the white‐breasted nuthatch (the great nuthatch *S. magna* ; Pasquet et al. 2014), nor for the wrentit (in a monotypic genus; no other members of the taxonomic family Paradoxornithidae have been examined; Gill et al. 2024).

For both the red‐browed finch and the Azores and Eurasian bullfinches, the closest relative with measured sperm has a substantially shortened midpiece, suggesting that shortening of the midpiece may precede the evolution of the tadpole‐like phenotype (Birkhead et al. 2006; Rowe et al. 2025). The closest relative for the two bullfinches also shows dramatically short sperm compared to other close relatives (Birkhead et al. 2006; Lifjeld et al. 2013), while for the red‐browed finch, sperm length decreased only in the lineage with the tadpole‐like phenotype (Rowe et al. 2025). The relatively elongated head of the red‐browed finch and wrentit (compared to the bullfinches and white‐breasted nuthatch) may similarly represent an intermediate and perhaps transitory form of sperm, between typical songbird sperm and the more spherical tadpole shape. As pointed out by Birkhead et al. (2007), intermediate phenotypes would suggest that the phenotypic shift in sperm has not been truly saltatory, but rather via a gradual (though perhaps rapid) process. Sampling close relatives of the white‐breasted nuthatch and wrentit would be useful to better estimate the timing of the shift to the tadpole‐like phenotype and to further investigate possible transitory phenotypes.

### 4.3 Ontogeny and Mechanism

According to the neoteny hypothesis, the tadpole‐like sperm phenotype arose due to a disruption in spermatogenesis that prevents these cells from reaching the typical fully mature phenotype (Birkhead et al. 2006). At the genomic level, this disruption could originate with mutations in a regulatory protein or region that controls the late stages of spermatogenesis, and it will be interesting to learn whether similar mutations are at work in all the species with tadpole sperm. At the hormonal level, we can hypothesize that low testicular testosterone levels may play a role in the formation of this phenotype. Low testicular testosterone interferes with spermatogenesis in mammals, including preventing round spermatids from developing into elongated spermatids (Walker 2011). These species appear likely to produce little testicular testosterone because they have small testes, and testosterone is produced by the Leydig cells of the testes (Walker 2011). Contrary to this prediction, the white‐breasted nuthatch has comparable circulating testosterone levels and elevated testicular expression of enzymes involved in testosterone production compared to other species (Schuppe and Fuxjager 2019; Schuppe et al. 2022). We are unaware of literature on the other species with tadpole‐like sperm.

An alternate hypothesis is that high levels of inbreeding during severe historical population bottlenecks could cause the shift to tadpole‐like sperm, since inbreeding is known to cause sperm abnormalities in endangered species (Durrant et al. 2010). This hypothesis has been refuted in the Eurasian bullfinch and red‐browed finch, which do not show evidence of restricted genetic diversity (Durrant et al. 2010; Rowe et al. 2025). Rigorous comparisons of genetic variation are not available for white‐breasted nuthatches or wrentits, but the available evidence does not suggest striking inbreeding in either species. Nucleotide diversity (*π*) in intronic markers in white‐breasted nuthatch populations ranged from 0.00123 to 0.00255 (Walstrom et al. 2012), similar to three other passerine species tested with an overlapping set of intronic markers (great reed warbler *Acrocephalus arundinaceus* , 0.0012, blue tit *Cyanistes caeruleus* 0.0018, and collared flycatcher *Ficedula albicollis* 0.0024; Backström et al. 2008). The wrentit shows typical levels of microsatellite diversity (Delaney et al. 2010) at microsatellite loci developed for Passerines in general (Richardson et al. 2000). These estimates reflect more recent diversity and may not account for ancient bottlenecks.

### 4.4 Adaptive Explanations

Similar to the red‐browed finch and the Eurasian and Azores bullfinches, the white‐breasted nuthatch and wrentit have traits consistent with low female promiscuity and therefore weak post‐copulatory sexual selection, based on several lines of evidence (Birkhead et al. 2006; Lifjeld et al. 2013; Rowe et al. 2025). Females are less promiscuous in species with long‐term pair bonds, high male investment during nest building and incubation, a sedentary lifestyle (i.e., no long‐distance migration, although the migration effect differs among phylogenetic studies), and where males have relatively small testes and high variation in sperm morphology (Calhim et al. 2007; Kleven et al. 2008; Immler et al. 2008; Lifjeld et al. 2010, 2019; Valcu et al. 2021). We emphasize that directly testing extra‐pair paternity levels in these species is important to assess female promiscuity levels, particularly since female promiscuity appears to respond very rapidly to changing ecological conditions (Brommer et al. 2010). If sperm quality were too low in species with tadpole‐like sperm, such that a substantial proportion of males were infertile, females could be expected to begin mating promiscuously (e.g., Hasson and Stone 2009).

Natural selection on sperm phenotype may dominate in species without strong sexual selection, and it has been hypothesized that the tadpole‐shaped sperm may be less costly to produce (Birkhead and Immler 2007). This cost reduction could be direct if producing larger sperm cells and/or elaborate structures on these cells is costly, as implied by Birkhead and Immler (2007), or it could be indirect. For example, there may be a cost to maintaining high circulating testosterone levels in species with long‐term pair bonds, high paternal investment, and no long‐distance migration (Hau et al. 2008); the tadpole‐like sperm form may allow males to produce fully functional sperm despite low testicular testosterone. Additionally, this phenotype could be a response to changes in mitochondrial function due to release from pressures related to oxidative stress from burning fat during long‐distance migration (McWilliams et al. 2021). Indirect costs of sperm production may be a more likely hypothesis, since direct cost minimization might be expected to drive the production of few, high‐quality sperm rather than more sperm of lower quality, so long as sperm are numerous enough to overcome the diluting effect of the relatively large volume of the female reproductive tract (Immler et al. 2011).

Possible alternative adaptive hypotheses include changes in the egg or the female reproductive tract that drive sperm shape. Coevolution with the egg membrane has been supported preliminarily in two of the putatively neotenous mammals (greater bandicoot rat and spinifex hopping mouse; McGregor et al. 1989; Bauer and Breed 2006; Dorman et al. 2014) but has not been tested in birds. Tight correlations are observed between the shape of sperm and of female sperm storage organs across species (Pitnick et al. 2009), and evolutionary path analysis within Passeriformes suggests that sperm form follows the form of female sperm storage tubules rather than vice versa (Briskie et al. 1997). Female sperm storage organs in zebra finches ( *Taeniopygia guttata* ) have a constricted opening, which plausibly may be more easily navigated by a helically shaped sperm (Mendonca et al. 2019). Whether and why female sperm storage organ length or shape has changed is unknown for most of the songbird species with tadpole‐like sperm. This hypothesis is not supported in the Eurasian bullfinch, where the single sampled female had sperm storage organs about six times longer than the species' sperm (Birkhead et al. 2006; sperm storage organs in most songbird species are two to three times longer than conspecific sperm; Briskie and Montgomerie 1992). Moreover, in captivity, Eurasian bullfinch eggs can be fertilized by sperm of species with typical sperm, suggesting that cryptic female choice against the typical sperm morphology must be weak (Birkhead and van Balen 2007). Finally, helical sperm heads have been linked to the viscosity of the fertilization environment in other animal taxa (Wang et al. 2021), suggesting that changes in the viscosity of the fluids in the female reproductive tract could be a selective factor.

It is further possible that the tadpole‐like sperm phenotype is adaptively neutral. This hypothesis is supported by the observation that relaxed purifying selection rather than directional selection elevates the rates of evolution of male‐reproductive genes in murine rodent species with low post‐copulatory sexual selection (including two species with putative neotenous phenotypes; Kopania et al. 2025). The tadpole‐like phenotype could be even weakly maladaptive and have been swept to fixation in songbird species due to linkage to an allele under strong selection (i.e., genetic hitchhiking; Rowe et al. 2025). This hypothesis remains to be tested once the relevant genomic regions have been identified.

Adaptive hypotheses about this phenotype may be more relevant for explaining its persistence over time rather than its emergence (which may be simply due to rare mutations). As such, the fact that many species have similar life‐history traits but typical sperm morphology is not strong evidence against these hypotheses. In contrast, discovering tadpole‐like sperm in species with higher post‐copulatory sexual selection and/or a different suite of ecological traits would allow us to reject some or all of the above hypotheses. Our current phylogenetic tests of the association between tadpole‐shaped sperm and life‐history traits are only preliminary and likely lack statistical power. That said, we find that tadpole‐shaped sperm is associated, at the level of taxonomic families, with low post‐copulatory sexual selection (low extra‐pair paternity and relatively small testes) and with shorter distance migration or sedentary lifestyles.

## 5 Conclusions

We present data suggesting two novel instances of dramatic shifts to tadpole‐like sperm phenotypes in songbirds. The tadpole‐like sperm phenotype is strikingly similar among the five bird species where it is known, with parallels to sudden evolutionary changes in sperm body plan in mammals, including a loss of components that provide structural support to the flagellum. This similarity raises the question of whether the underlying genetic, genomic, and developmental causes of the tadpole‐like phenotype are similarly parallel, or whether diverse types of mutations, rearrangements, or alterations to gene regulation simply result in a very similar sperm phenotype. Further study on the ecology and physiology of these species, as well as sampling sperm in species where female promiscuity levels are disassociated from pair‐bond duration, migratory status, and paternal investment, will help to distinguish which selective forces are driving this phenotype.

## Author Contributions

**Emily R. A. Cramer:** conceptualization (lead), formal analysis (equal), funding acquisition (equal), investigation (equal), writing – original draft (equal), writing – review and editing (equal). **Gaute Grønstøl:** investigation (equal), visualization (equal), writing – review and editing (equal). **Phred M. Benham:** investigation (equal), project administration (equal), writing – review and editing (equal). **Carla Cicero:** investigation (equal), project administration (equal), writing – review and editing (equal). **Rauri C. K. Bowie:** funding acquisition (equal), investigation (equal), investigation (equal), project administration (equal), project administration (equal), resources (equal), resources (equal), writing – review and editing (equal), writing – review and editing (equal). **Daniel J. Tobiansky:** funding acquisition (equal), investigation (equal), project administration (equal), resources (equal), writing – review and editing (equal). **Jan T. Lifjeld:** conceptualization (supporting), funding acquisition (equal), project administration (equal), resources (equal), writing – review and editing (equal).

## Conflicts of Interest

The authors declare no conflicts of interest.

## Data Availability

Data are available at Data Dryad, DOI: https://doi.org/10.5061/dryad.p8cz8wb1t.
